# Identification and characterization of Aβ peptide interactors in Alzheimer’s disease by structural approaches

**DOI:** 10.3389/fnagi.2014.00265

**Published:** 2014-10-09

**Authors:** Keith D. Philibert, Robert A. Marr, Eric M. Norstrom, Marc J. Glucksman

**Affiliations:** ^1^Department of Biochemistry and Molecular Biology, Chicago Medical School, Rosalind Franklin University of Medicine and ScienceNorth Chicago, IL, USA; ^2^Department of Neuroscience, Chicago Medical School, Rosalind Franklin University of Medicine and ScienceNorth Chicago, IL, USA; ^3^Department of Biological Sciences, DePaul UniversityChicago, IL, USA

**Keywords:** Alzheimer’s disease, Aβ peptide, EP24.15, transthyretin, Proteomics

## Abstract

Currently, there are very limited pharmaceutical interventions for Alzheimer’s disease (AD) to alleviate the amyloid burden implicated in the pathophysiology of the disease. Alzheimer’s disease is characterized immunohistologically by the accumulation of senile plaques in the brain with afflicted patients progressively losing short-term memory and, ultimately, cognition. Although significant improvements in clinical diagnosis and care for AD patients have been made, effective treatments for this devastating disease remain elusive. A key component of the amyloid burden of AD comes from accumulation of the amyloid-beta (Aβ) peptide which comes from processing of the amyloid precursor protein (APP) by enzymes termed secretases, leading to production of these toxic Aβ peptides of 40–42 amino acids. New therapeutic approaches for reducing Aβ are warranted after the most logical avenues of inhibiting secretase activity appear less than optimal in ameliorating the progression of AD.Novel therapeutics may be gleaned from proteomics biomarker initiatives to yield detailed molecular interactions of enzymes and their potential substrates. Explicating the APPome by deciphering protein complexes forming in cells is a complementary approach to unveil novel molecular interactions with the amyloidogenic peptide precursor to both understand the biology and develop potential upstream drug targets. Utilizing these strategies we have identified EC 3.4.24.15 (EP24.15), a zinc metalloprotease related to neprilysin (NEP), with the ability to catabolize Aβ 1–42 by examining first potential *in silico* docking and then verification by mass spectrometry. In addition, a hormone carrier protein, transthyreitin (TTR), was identified and with its abundance in cerebrospinal fluid (CSF), found to clear Aβ by inhibiting formation of oligomeric forms of Aβ peptide. The confluence of complementary strategies may allow new therapeutic avenues as well as biomarkers for AD that will aid in diagnosis, prognosis and treatment.

## Introduction

Alzheimer’s disease is a terrible neurodegenerative disorder affecting the elderly resulting in progressive mental decline with profound effects on memory. Accumulating evidence indicates that the failure to clear Aβ from the brain is a primary step in the pathology of sporadic Alzheimer’s disease (AD; Crews and Masliah, 2010). Many of the most potent gene polymorphisms that confer risk for AD affect clearance of Aβ (Bu, 2009; Kim et al., 2013; Reitz et al., 2013). This is underscored by the finding that individuals with AD are deficient in Aβ clearance while the rate of Aβ production is unaltered (Mawuenyega et al., 2010). It has long been suspected that the disruption of Aβ catabolism is a critical factor in Aβ accumulation (Mukherjee and Hersh, 2002).

Endopeptidases are peptidolytic enzymes that cleave proteins at internal peptide bonds, and are extremely important in synthetic and degradative neuropeptide metabolism. Direct proteolysis by endopeptidases is a major pathway of Aβ clearance and there are several enzymes that can cleave this peptide (Miners et al., 2011; Nalivaeva et al., 2012). The usual suspects include angiotensin converting enzyme (ACE), endothelin converting enzymes (ECE), neprilysin (NEP) and insulin degrading enzyme (IDE). While mice genetically deficient in ACE did not show elevated levels of Aβ (Eckman et al., 2006; Hemming et al., 2007); ACE may still be relevant in the human system as mutations in the ACE gene have been linked to increased risk of AD (Bertram and Tanzi, 2008). Mice deficient in ECE-1 or NEP show elevated levels of the Aβ peptide (Iwata et al., 2001; Eckman et al., 2003, 2006; Madani et al., 2006; Rodriguiz et al., 2008; Walther et al., 2009), and some polymorphisms in these genes have been associated with risk of developing AD (Marr and Spencer, 2010; Pacheco-Quinto et al., 2013). Deficiencies in IDE also produce elevated levels of Aβ in mice (Farris et al., 2003; Miller et al., 2003) and mutations have been linked to AD risk (Kim et al., 2007; Zhang et al., 2013). More recently, Neprilysin-2 (NEP2), which has been demonstrated *in vivo* to control Aβ levels by cleaving Aβ between amino acids Val18 and Leu17 similar to ECE-1 (Huang et al., 2008), is reduced in association with AD (Huang et al., 2008, 2012; Hanson et al., 2010; Hafez et al., 2011).

In addition to these endopeptidases, there are many other factors that may contribute to Aβ clearance including enzymes and carrier proteins (reviewed in Miners et al., 2011; Nalivaeva et al., 2012; Leissring and Turner, 2013) (also see Marr and Hafez, 2014 in press in this issue). Neuromics technology is the proteomics of the neural systems and the brain in particular and has advanced such that both known and novel interactors can be identified without bias. The ability to isolate *in vivo* protein complexes under close to physiological conditions reveals the complexity of interactions that can be considered in the context of systems biology (Gingras et al., 2005; Collins and Choudhary, 2008). The first interaction we have examined was the role of novel proteases involved in Aβ clearance.

We have sought to discover new activities that can mitigate the effects of Aβ by utilizing this peptide as a substrate for catabolism. One such peptidase has been EP24.15 (thimet oligopeptidase, THOP; EC 3.4.24.15), an amyloid precursor protein (APP) interacting protein that has been chronicled in the context of AD. EP24.15 is a soluble, 77 kD metalloendopeptidase with extracellular peptide processing/degrading activity in the family of zinc metallopeptidases that can promote the clearance of Aβ. In the central nervous system EP24.15 is involved in the catabolism of bioactive peptides including the generation of enkephalins, and processing of other neuropeptides such as bradykinin, gonadotropin releasing hormone and nociceptive peptides (Tisljar, 1993; Kim et al., 2003).

Early literature was somewhat conflicting explicating the role of the EP24.15 metalloenzyme in the etiology of AD. This enzyme was originally erroneously thought to be the β-secretase activity initiating the processing of the APP into Aβ (Papastoitsis et al., 1994) prior to the discovery of the beta amyloid cleaving enzyme-1 (BACE-1). The EP24.15 gene is located at a chromosomal locus that is close to a region involved with early onset AD (Meckelein et al., 1996). EP24.15 expression has been shown to inhibit the toxicity of Aβ *in vitro* and to colocalize with Aβ plaques in an APP transgenic mouse model of AD (Pollio et al., 2008). These studies indicate that EP24.15 is potentially involved in the pathogenesis of AD.

In addition to enzymes, carrier proteins play an important role in Aβ clearance. One of these factors, transthyretin (TTR), was first identified in a screen of cerebrospinal fluid (CSF) proteins which were found to be capable of inhibiting Aβ fibrillogenesis (Schwarzman et al., 1994). Transthyretin is a homotetramer consisting of four identical subunits of approximately 14 kD each with detailed atomic structure elucidated by X-Ray crystallography (Hörnberg et al., 2000). Transthyretin is known to bind thyroid hormones and retinol binding protein in serum (Schreiber and Richardson, 1997) suggestive of a role as a hormone carrier protein with albumin-like qualities. Plasma TTR is primarily synthesized in the liver while the choroid plexus synthesizes TTR which is found abundantly in CSF making up ~25% of total CSF proteins (Aldred et al., 1995). Transthyretin can itself form amyloid under conditions in which the protein is monomerized and partially unfolded (Kelly et al., 1997), however at neutral to basic pH, it is extremely stable (Hammarström et al., 2002).

Recent attention has focused on brain and CSF TTR as a potential carrier for the clearance of Aβ peptide. Studies have shown that expression of human TTR in *C. elegans* can rescue behavior and pathology associated with expression of human Aβ in muscle (Link, 1995). Moreover, transgenic mice that express AD-related mutations exhibited accelerated rates of Aβ deposition concomitant with hemizygous deletion of the TTR gene (Choi et al., 2007) whereas overexpression of TTR suppressed pathological and behavioral abnormalities associated with a transgenic model of AD (Buxbaum et al., 2008). Recent evidence suggests a potential positive-feedback regulation mechanism of APP whereby increased generation of the APP intracellular domain (AICD) leads to upregulation of Aβ clearing transcripts for TTR and NEP (Kerridge et al., 2014).

In this work, we implement the integration of biophysical results from molecular modeling, mass spectrometry, X-ray crystallography, and Surface Plasmon Resonance (SPR) with methods most often used to identify components of Aβ clearance. We provide two examples, EP24.15, a neuropeptide processing enzyme, and TTR, a carrier protein expressed in the CSF and plasma that transports thyroid hormones and retinol. In this manner, the progression from discovery to confirmation and then validation can begin to vet interactors with Aβ for future therapeutics to alter the equilibrium towards diminishing the amyloid burden present in AD.

## Materials and methods

### Expression and purification of Aβ-interacting proteins

#### EP24.15

Wild type EP24.15 was expressed and purified as previously described (Cummins et al., 1999). In brief, BL21 Gold competent *E. coli* (Agilent, La Jolla, CA), transformed with pGEX2T-EP24.15, was grown to OD_600_ = 0.8 and protein expression was induced with 0.5 mM IPTG for 5 h at 16°C. Pelleted cells were resuspended in 100 mM Tris-HCl (pH 7.4) containing protease inhibitor cocktail (Sigma, St Louis, MO) and passaged five times through a French press homogenization cell followed by centrifugation for 15 min at 14,000 ×g at 4°C. The clarified extract was bound for 4 h at 4°C with rotation to Glutathione-Sepharose 4B (GE Healthcare Life Sciences, Piscataway, NJ). The glutathione-S-transferase tag was cleaved with thrombin (MP Biomedicals, Santa Ana, CA) at 6 U/ml bed volume overnight at room temperature. Wild type enzyme was eluted with 10 volumes of 100 mM Tris-HCl (pH 7.4), concentrated with 30 kDa MWCO Amicon Ultra centrifugal filters (Millipore, Billerica, MA) and the final protein concentration adjusted to 5 mg/ml following Bradford protein determination (Bio-Rad, Hercules, CA) and aliquoted for storage at −80°C until use. Sodium dodecyl sulfate acrylamide gel electrophoresis (SDS-PAGE) was used to confirm enzyme purity and quality.

#### TTR

The TTR open reading frame was cloned from a human cDNA library (Molecular Biology and Genetics Core, University of Chicago). For expression and purification, the eukaryotic signal sequence was deleted and an N-terminal 6-histidine tag was cloned in its place. This construct was subcloned into the pProEx bacterial expression vector and expressed by induction with 0.1 mM IPTG in the Rosetta (DE3) bacterial strain (EMD Millipore) for 16 h at 25°C, conditions which preliminary expression experiments had demonstrated to be optimal for expression and solubility of TTR. Analysis of the crude bacterial lysate by Coomassie staining and Western immunoblot analyses indicated robust expression and solubility of TTR. Based on this initial expression, a large scale purification was performed in which 4 liters of media were inoculated and allowed to express for 24 h. Pelleted bacteria were resuspended in 250 mL lysate buffer (20 mM Tris-Cl pH 7.7, 100 mM NaCl, 100 µM PMSF, 0.1 mg/mL lysozyme), sonicated in lysate buffer and cleared of debris with a low speed spin. The supernatant fraction was further centrifuged at 100,000 ×g for 1 h and the supernatant containing soluble TTR was collected. Transthyretin was purified from this fraction, first by capture of the 6-His moiety on Ni-nitrilotriacetic acid (NTA) resin, followed by anion exchange chromatography and then precipitation of contaminants at 37°C (Reixach et al., 2008). The final TTR preparation using these methods was approximately 95% pure as judged by Coomassie staining of SDS-PAGE.

Since TTR is normally present as a homotetramer, samples were submitted to native gel electrophoresis to assess the relative mass. Transthyretin tetramers, with a calculated weight of 60 kD with the His-tag, could be detected on silver-stained native PAGE gels migrating just below bovine serum albumin (66 kD), indicating that TTR was indeed tetrameric (Figure 4). The elution profile of TTR preps submitted to size exclusion chromatography indicated a size consistent with the previous results when calculated against multiple standards (data not shown). Analysis of preparations was performed using SDS-PAGE and subsequent Western immunoblotting.

**Figure 1:**
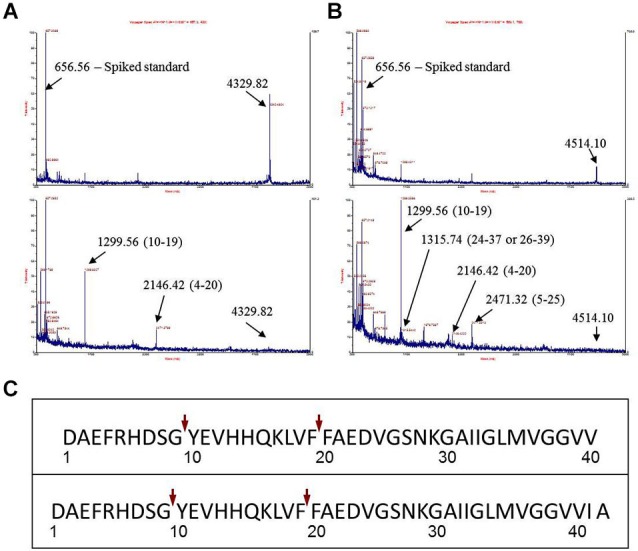
**Identification of major Aβ fragments by mass spectrometry after incubation with EP24.15**. **(A)** Mass Spectrum Aβ 1–40, **(B)** Mass Spectrum Aβ 1–42, **(C)** Composite Major Cleavage Sites: Aβ 1–42.

**Figure 2:**
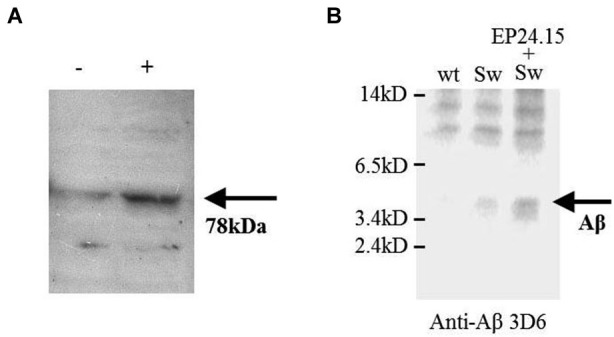
**The effect of EP24.15 overexpression on Aβ metabolism when co-transfected with cells expressing the “Swedish” mutation**. **(A)** M17 neuroblastoma cells transfected with APPsw and either mock (Tx−) or EP24.15 (Tx+) and probed with an antibody for EP24.15. Western immunoblotting on a 10% SDS-PAGE gel. **(B)** M17 neuroblastoma cells transfected with either vector alone, APPsw or APPsw + EP24.15 and Aβ and processed fragments probed with an N-terminal antibody for Aβ. Western immunoblotting on a 16.5% SDS-PAGE gel.

**Figure 3:**
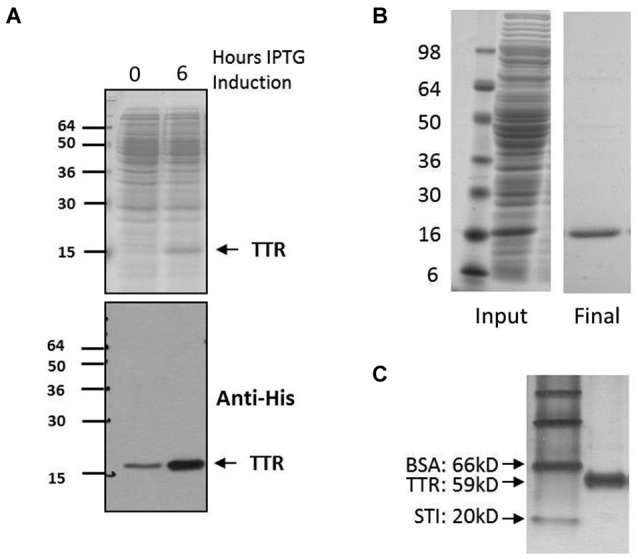
**Expression and purification of TTR. (A)** Expression of TTR. Top, 12% acrylamide SDS-PAGE and Coomassie stain of pre- and post-induction using IPTG. Bottom, Western blot of same samples with specific detection of TTR using anit-His. **(B)** Purification of TTR using FPLC and Ni^2+^ chromatography yielded pure TTR of approximately 95% purity as indicated by 12% acrylamide SDS-PAGE and Coomassie staining. **(C)** Purified sample run on 10% acrylamide Native-PAGE and stained by silver staining. Relative migration rate of 59 kD indicative of tetramer assembly was observed by native-PAGE whereas monomeric TTR is detected at approximately 15 kD by denaturing electrophoresis **(A** and **B)**.

**Figure 4:**
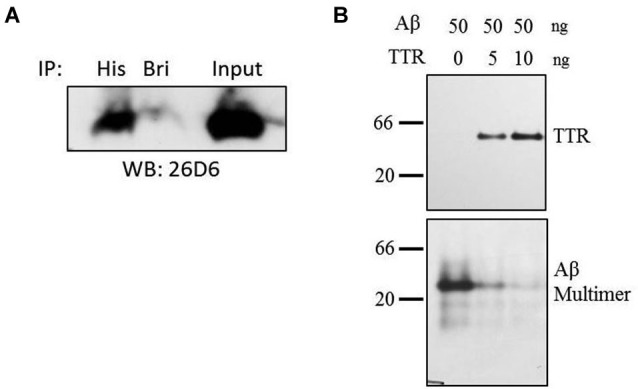
**Co-immunoprecipitation and inhibition of Aβ multimer formation. (A)** Purified samples of TTR and Aβ were incubated in HBS at equimolar ratios of 1 µM for 8 h at 4°C after which complexes were captured using anti-His or unrelated (anti-BRI) antibody. Amyloid-beta was detected using monocolonal antibody 26D6 followed by 16.5% acrylamide tris-tricine SDS-PAGE. **(B)** Amyloid-beta multimers form in the absence of TTR, but addition of TTR to Aβ solution inhibits multimer formation as assessed by native PAGE. Samples were run on 10% acrylamide gels using native-PAGE at pH 8.8. Numbers to the left indicate relative molecular weight.

#### Aβ cleavage assay

To determine if EP24.15 cleaves Aβ 1–40 or Aβ 1–42 as potential substrates, we incubated 300 µM Aβ peptide or neurotensin (positive control) in the presence of 5 ng wild type EP24.15 in 25 mM Tris-HCl (pH 7.4), 125 mM NaCl, 1 mM DTT, and 2% DMSO for various times up to 1 h at 37°C. At the indicated times, a 4 µl aliquot was removed from each reaction, diluted with 12 µl of 10 mg/ml α-Cyano-4-hydroxycinnamic acid (CHCA, Sigma) in 60% acetonitrile/0.1% formic acid (Optima LCMS grade; Fisher Scientific, Pittsburgh, PA).

#### Mass spectrometry and bioinformatics

Two microliters of the cleavage assay reaction was then spotted on a MALDI plate. The dried samples were analyzed using a Voyager DE STR mass spectrometer (AB Sciex, Framingham, MA) and the resulting peaks were matched to Aβ fragments using Expasy’s FindPept tool^1^. The following Voyager DE settings were used for all samples: 200 laser shots per spot at 1870 J, 350–4700 Da mass window with 350 Da low mass gate.

#### Transfection of EP24.15 into cell lines and western immunoblotting

Human recombinant EP24.15 was subcloned into a pcDNA3 vector containing a high-level transcription system (Invitrogen). This construct was cotransfected into a M17 neuroblastoma cell line already stably expressing APP containing the “Swedish” mutation at the beta secretase site. The EP24.15 antibody was used as described (Ferro et al., 1999; Massarelli et al., 1999) for Western immunoblotting. For detection of Aβ, an N-terminal antibody 3D6 (Elan Pharmaceuticals, South San Francisco, CA) was utilized for detecting Aβ peptide.

#### TTR and Aβ co-immunoprecipitation

Because of the high insolubility of Aβ 1–42 and its propensity for aggregation, all experiments were performed using Aβ 1–40 peptides purchased from American Peptide Company. For co-immunoprecipitation experiments, purified TTR and Aβ 1–40 peptides were combined in equimolar ratios in HEPES buffered saline (50 mM HEPES pH 8.0, 150 mM NaCl) and allowed to form complexes at 4°C for 8 h. Complexes were captured by addition of rabbit anti-6His His-tag antibody (GenScript cat#A00174-100) overnight at 4°C after which the antibody was immobilized with protein A Sepharose beads. In parallel, control preparation included an antibody directed against at target unrelated to Aβ, the protein BRI (Kim et al., 2002). Beads were washed five times with HEPES buffered saline containing 0.05% Triton X-100 and complexes were released by boiling in Laemmli sample buffer prior to analysis by PAGE and Western blot. Amyloid-beta was then detected using mouse monoclonal antibody 26D6 which binds to the aa 1–12 of the Aβ peptide. Anti-BRI and 26D6 antibodies were a kind of gift of Sangram Sisodia (University of Chicago).

#### Western immunoblotting of TTR

Samples were submitted to SDS-PAGE and transferred to polyvinylidine fluoride membranes (Bio-Rad). Membranes were washed in phosphate buffered saline containing 0.1% Tween20 (PBST) and blocked in PBST/5% milk for 1 h. After blocking, membranes were incubated with primary antibodies for 3 h at room temperature in PBST/1% milk washed three times in PBST and incubated in HRP-conjugated secondary antibody (Thermo Scientific) in PBST/1% milk for 1 h. For detection, blots were washed four times in PBST followed by incubation in Super Signal West Pico chemiluminescent substrate (Thermo Scientific) and detected using a Bio-Rad XRS Chemidoc imager or autoradiography film (Thermo Scientific).

#### Surface plasmon resonance

Surface plasmon resonance measurements were made using a BIACORE3000 instrument (GE Healthcare Life Sciences). A preconditioned Sensor Chip NTA (GE Healthcare Life Sciences) was bound with Ni^2+^ solution at a speed of 20 µl/min. Tetrameric His-tagged TTR was immobilized by flow of a 20 µM solution in HBS-P buffer (10 mM HEPES pH 9.0, 0.15 M NaCl, 0.005% Tween20, 0.5% DMSO) over the charged chip. The Aβ used was dispersed into monomers from lyophilized aliquots using DMSO before dilution into SPR binding buffer. For each concentration of Aβ, the chip was stripped with imidazole and EDTA then recharged with TTR. All steps except for the Ni^2+^ binding step were performed in a parallel channel of the Sensor Chip, and these values were subtracted from the experimental channel to obtain Response Unit (R.U.) values. Due to variability in results, perhaps due to the hydrophobic nature of the Aβ peptide, the experiment was repeated three times independently. The data were analyzed using BiaEvaluation (Bio-Rad) software for curve fitting.

## Results

### Neuropeptide processing enzyme EP24.15

Utilizing the model of the structure of the empty form of EP24.15 (Research Collaboratory for Structural Bioinformatics—Protein Data Bank, entry 1S4B; Ray et al., 2004), Aβ 1–40 or Aβ 1–42 could potentially interact *in silico* upon “closing” around the substrate (data not shown) with subsequently cleaved peptide products that can be subjected to amino and carboxypeptidases for further degradation into smaller peptide fragments.

The first experimental confirmation of the putative Aβ degrading activity involved incubation of purified enzyme and Aβ 1–40 and 1–42 peptides in separate reactions. Cleavage reactions were performed at *t* = 0’ and 30’ with Aβ 1–40 and Aβ 1–42 as potential substrates (Figures 1A,B, respectively) under first order kinetics and then subjected to matrix assisted laser desorption ionization—time of flight (MALDI–TOF) mass spectrometry. Cleaved fragments of Aβ peptide Aβ 1–40 (Table 1) and Aβ 1–42 (Table 2), respectively were processed using the known sequence and their cleavage points mapped. Major cleavages are indicated by the red arrows (Figure 1C).

**Table 1:** **Cleavage fragments of Aβ1–40**.

Peptide mass (Da)	Peptide fragment	Sequence
485.946	30–34	AIIGL
525.026	17–20	LVFF
672.661	27–33	NKGAIIG
**1299.56**	**10–19**	**YEVHHQKLVF**
2146.42	4–20	FRHDSGYEVHHQKLVFF
4329.82	1–40	DAEFRHDSGYEVHHQKLVFFAEDVGSNKGAIIGLMVGGVV

**Table 2:** **Cleavage fragments of Aβ1–42**.

Peptide mass (Da)	Peptide fragment	Sequence
490.214	21–25	FAEDVG
525.026	17–20	LVFF
662.628	13–17	HHQKL
**1299.56**	**10–19**	**YEVHHQKLVF**
1315.74	26–39	SNKGAIIGLMVGGVVI
2146.42	4–20	FRHDSGYEVHHQKLVFF
2471.32	5–25	RHDSG YEVHHQKLVF FAEDVG
4514.10	1–42	DAEFRHDSGYEVHHQKLVFFAEDVGSNKGAIIGLMVGGVVIA

Human recombinant EP24.15 was cotransfected into a model of Aβ processing (Cai et al., 1993) consisting of human M17 neuroblastoma cells with full-length wild type APP or APP containing the hyperprocessed “Swedish” mutation (KM 595/596 NL). The amount of soluble products of Aβ ~4 kD and smaller was monitored via gel electrophoresis on a 16.5% gel. Overexpression of EP24.15 was achieved in the APP “Swedish” mutation background (Figure 2A) and the appearance of smaller products was enhanced as well as with the stably transfected EP24.15 when blots were probed with the N-terminally directed antibody (Figure 2B) in the background of the “Swedish” mutation compared to the “Swedish” mutation alone.

### Transthyretin

The expression of TTR in bacteria was assessed by comparing pre and post induction with IPTG (Figure 3A). After isolation from bacteria, lysates were purified utilizing fast performance protein chromatography (FPLC) with affinity chromatography targeting the His tag on TTR (Figure 3B). In solution, the complexes assemble into a higher order tetramer as indicated by Native Gel Electrophoresis (Figure 3C).

Direct interactions between TTR and Aβ were implicated by co-immunoprecipitation (Co-IP) in a purified system containing only TTR and Aβ. Thus, co-IP experiments were performed in solution containing purified samples of both TTR and Aβ. Transthyretin and Aβ were combined in equimolar ratios in HEPES buffered saline and allowed to complex for 8 h at 4°C after which an anti-6-Histidine antibody or an unrelated antibody (anti-BRI antibody) was added to the solution followed by overnight incubation. After a pulldown using Protein A beads, Aβ was easily detectable by Western blot in the lane containing the anti-His antibody (Figure 4A). A small amount of residual Aβ was detected in the control lane, likely due to the fact that Aβ’s hydrophobic properties allow for non-specific interactions with the control antibody as well as the beads. Thus, TTR and Aβ can interact in purified solutions indicating a direct, binary interaction.

Since TTR binds to Aβ in solution, formation of multimeric Aβ (and by extension, insoluble fibrillar Aβ), would be inhibited by its presence. To test this, purified TTR was incubated with Aβ for 16 h at room temperature, and the formation of multimeric Aβ species was assayed by native PAGE on Tris-glycine pH 8.8 gels followed by transfer to polyvinylidene difluoride (PVDF) and Western blotting. With increasing TTR concentrations (Figure 4A, top panel), Aβ multimer formation was reduced (Figure 4B, bottom panel). Monomeric species are not detectable on this native gel, but separate analyses indicated that proteolytic activity was not present (data not shown). Thus, TTR was found to multimerize and inhibit the formation of high molecular weight oligomeric Aβ species in a purified *in vitro* system.

**Figure 5:**
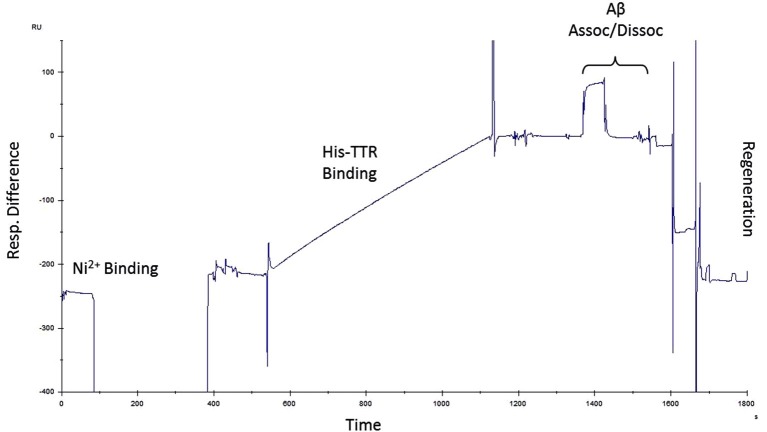
**Surface plasmon resonance protocol**. Example of a single run for SPR experiments. Ni^2+^ was used to charge NTA chips followed by His-TTR binding to Ni^2+^–NTA. Amyloid-beta was then passed over the immobilized TTR during which association and dissociation periods were recorded. After each run, the chip was regenerated and the protocol was repeated with another concentration of Aβ.

Next, we sought to determine the affinity of Aβ for TTR. To accomplish this, we performed SPR experiments on purified TTR and Aβ. Since the TTR purified for these experiments contains a 6-His tag, we first utilized an NTA chip which coordinates Ni^2+^ and allows binding of the 6-His tag. For these experiments, NTA Sensor Chips were charged with Ni^2+^, bound with the TTR, and Aβ was passed over the immobilized ligand. To account for non-specific binding to the surface, all analyte samples are also passed over a surface without ligand for a reference value which is subtracted from the ligand channel to yield a Response Difference value. Each step results in a change in the resonance units (RU) monitored as a function of time. A typical experiment is depicted on the sensorgram (Figure 5).

**Figure 6:**
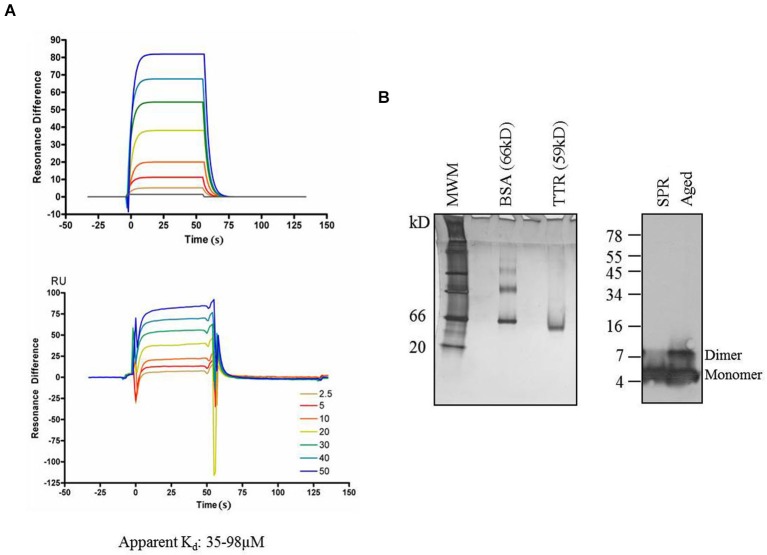
**Transthyretin and Aβ. (A)** Surface Plasmon Resonance profiles of Aβ binding to immobilized TTR. Top—fits to data on bottom. Experiment was repeated three times with apparent kD values ranging from 35–98 µM. **(B)** Analysis by 10% acrylamide Native-PAGE and silver stain of TTR samples used for SPR at completion sample input runs indicated that TTR was stably present as a tetramer and was detected at the expected relative mass of 59 kD. Similarly, Aβ used in SPR experiments maintained its monomer form throughout the experiment, and no SDS-resistant multimers were apparent by Western blot from samples taken at the completion of the experiment, though multimers are apparent in Aβ aged for 72 h in the same buffer as the TTR experiments. MWM = molecular weight markers.

Using this paradigm, Aβ concentrations ranging from 2.5–50 µM were injected for 30 s followed by buffer alone for 30 s to measure the association/dissociation kinetics of Aβ to immobilized TTR (O’Shannessy et al., 1994). After each concentration of Aβ, the chip was regenerated using imidazole and EDTA and the cycle is repeated. An equal amount of TTR is bound for each subsequent assay and three separate, independent experiments were performed. The best fits using 1:1 Langmuir binding formulas yielded an apparent kD within a similar range of 38 µM, 53 µM, and 98 µM for the three experiments (Figure 6A), consistent with previous reports of TTR-Aβ affinities in which Aβ was immobilized on the SPR chips and TTR was injected (Li et al., 2013).

To ensure that the TTR used in our experiments remained in a tetrameric state, samples of TTR were removed from those used in experiments at the conclusion of the SPR run. When fractionated on native gels and stained with silver nitrate, only a single band migrating at a M_r_ of ~60 kD was detectable and no bands were detectable at the monomeric mass of 15 kD. Thus, TTR was indeed present in the functional tetrameric state throughout the experiment. Another potential concern for the SPR experiment is the propensity of Aβ to form aggregation structures in solution. To ensure that Aβ remained in a non-aggregated state throughout the experiment, samples of the solution containing Aβ were taken at the completion of the experiments and analyzed for the presence of high-molecular weight structures using SDS-PAGE (Rosen et al., 2010). No SDS-resistant Aβ multimers were detectable when fractionated on tris-tricine SDS-PAGE gels (Figure 6B—left lane) compared to an aged Aβ sample incubated in the same buffer for 72 h in which SDS-resistant dimers are present (Figure 6B—right lane).

## Discussion

By using a panoply of biophysical technology we can assess interactors of Aβ peptide leading to its potential clearance and reduce neuropathology associated with AD. We demonstrate two potential interacting proteins that can affect the distribution of neurotoxic Aβ: the neuropeptide processing enzyme EP24.15 and carrier protein TTR.

EP24.15 has long been considered a factor in the etiology of AD albeit in mistaken contexts. Prior to the discovery of the transmembrane, aspartic acid protease, BACE, EP24.15 was thought to be the β-secretase activity initiating the processing of the APP into Aβ upon purification from human AD brains (Papastoitsis et al., 1994) or from platelets and megakaryocytes (Abraham et al., 1999). Subsequently, this group hypothesized that the expression of EP24.15 induced Aβ degrading activity *in vitro* through the stimulation/activation of an Aβ-degrading serine proteinase activity (Yamin et al., 1999) from conditioned medium from antisense transfected SKNMC neuroblastoma cell line. An interaction with alpha 1-antichymotrypsin, elevated in AD brain, and upregulation of serine proteases by EP24.15 was hypothesized. However, our data supports a role for EP24.15 in direct degradation of Aβ (Figure 1) that may occur concomitantly with the upregulation of serine proteases. Earlier work with synthetic peptides that spanned the beta secretase site at the amino terminus of Aβ (not the entire Aβ peptide) demonstrated cleavage by EP24.15, but this was not substantiated *in vivo* with APP degradation (Papastoitsis et al., 1994; Thompson et al., 1997).

By measuring the conversion of somatostatin 28 to somatostatin 1–14 and the degradation of substance P (both substrates of EP24.15), as poor, less specific surrogates for EP24.15 in the temporal cortex, it was implied that regulation of EP24.15 was significantly altered in AD (Waters and David, 1995; Waters and Davis, 1997). When the activity of a panel of exo- and endopeptidases was surveyed in the frontal and parietal cortices as well as cerebellums of patients diagnosed with AD there were reductions observed with several enzymes including EP24.15 (Ichai et al., 1994).

EP24.15 expression has been shown to inhibit the toxicity of Aβ *in vitro* (Pollio et al., 2008). In older transgenic animals, EP24.15 protein was increased in the hippocampus, supportive of a role in Aβ clearance though there was no attributable mechanism. Interestingly, with regards to immunocytochemistry, EP24.15 has been shown to localize in astrocytes and microglia with double staining with glial fibrillary acidic protein and CD11b markers respectively (Arif et al., 2009).

While, direct proteolytic degradation of Aβ is a critical part of the clearance of this peptide, other modes of clearance are also important. This includes interactions that affect the aggregation of Aβ, as this is believed to be an essential step in its neurotoxicity. An interaction between TTR and Aβ is posited to occur based on evidence *in vivo* that TTR may play a role in Aβ clearance. Co-immunoprecipitation of TTR and Aβ has been performed in neural tissues (Buxbaum et al., 2008). While it is apparent that TTR can affect Aβ fibrillization *in vivo*, direct biochemical characterization of a TTR/Aβ complex has only recently begun (Buxbaum et al., 2008; Li et al., 2013). Thus, we sought to demonstrate that TTR can inhibit the formation of oligomeric Aβ in systems containing only Aβ and TTR. Co-immunoprecipitation can also be performed *in vitro* and we further characterize a direct TTR/Aβ interaction by SPR. The results are consistent with a role for TTR in the clearance of Aβ from neuronal tissues. The affinities in the low micromolar range are consistent with previous reports for TTR/Aβ interactions (Li et al., 2013), and considering the high concentration of TTR in CSF and plasma, are likely biologically relevant for the binding and clearance of Aβ from interstitial fluids.

Most recently, there appears to be epigenetic regulation of both TTR and NEP, a peptidase closely related to EP24.15 (Kerridge et al., 2014). The AICD has been shown to bind to the NEP promoter upregulating transcriptional activation by histone deacetylases with a concomitant increase in enzyme activity causing a potential increase in Aβ clearance. Taken together, these data indicate that TTR and Aβ interact with low but detectable affinity in the micromolar range in purified solutions and that TTR can inhibit Aβ multimer formation consistent with findings in the literature. Both of these effects are predicted to be protective from AD pathology.

Utilizing approaches in the neuroma, such as identifying interacting proteins that bind to APP or its neurotoxic products, can aid in the development of effective pharmacological approaches for patients afflicted by AD. As potential biomarkers, these targets can aid in diagnosis and prognosis as well as treatment by increasing clearance of Aβ.

## Author contributions

Experiments were performed by Keith D. Philibert, Eric M. Norstrom and Marc J. Glucksman, studies were conceived and planned by Keith D. Philibert, Robert A. Marr, Eric M. Norstrom and Marc J. Glucksman and the manuscript was prepared by Keith D. Philibert, Robert A. Marr, Eric M. Norstrom and Marc J. Glucksman.

## Conflict of interest statement

The authors declare that the research was conducted in the absence of any commercial or financial relationships that could be construed as a potential conflict of interest.

## Footnotes

^1^http://web.expasy.org/findpept/
